# Androgen loss weakens anti-tumor immunity and accelerates brain tumor growth

**DOI:** 10.21203/rs.3.rs-4014556/v1

**Published:** 2024-03-29

**Authors:** Juyeun Lee, Yoon-Mi Chung, Lee Curtin, Daniel J. Silver, Yue Hao, Cathy Li, Josephine Volovetz, Ellen S. Hong, Jakub Jarmula, Sabrina Z. Wang, Kristen E. Kay, Michael Berens, Michael Nicosia, Kristin R. Swanson, Nima Sharifi, Justin D. Lathia

**Affiliations:** 1 Department of Cardiovascular and Metabolic Sciences, Lerner Research Institute, Cleveland Clinic, Cleveland, OH, USA; 2 Desai Sethi Urology Institute, Miller School of Medicine, University of Miami, Miami, FL, USA; 3 Sylvester Comprehensive Cancer Center, University of Miami; 4 Mayo Clinic, Mathematical NeuroOncology Lab, Precision Neurotherapeutics Innovation Program, Mayo Clinic, AZ, USA; 5 Department of Neurosurgery, Mayo Clinic, AZ, USA; 6 TGen, Translational Genomics Research Institute, Phoenix, AZ, USA.; 7 Medical Scientist Training Program, Department of Medicine, Case Western Reserve University, Cleveland, OH, USA; 8 Department of Molecular Medicine, Cleveland Clinic Lerner College of Medicine, Case Western Reserve University, Cleveland, OH, USA; 9 Department of Inflammation and Immunity, Lerner Research Institute, Cleveland Clinic, Cleveland, OH, USA; 10 Rose Ella Burkhardt Brain Tumor Center, Cleveland Clinic, Cleveland, OH, USA; 11 Case Comprehensive Cancer Center, Cleveland, OH, USA

## Abstract

Many cancers, including glioblastoma (GBM), have a male-biased sex difference in incidence and outcome. The underlying reasons for this sex bias are unclear but likely involve differences in tumor cell state and immune response. This effect is further amplified by sex hormones, including androgens, which have been shown to inhibit anti-tumor T cell immunity. Here, we show that androgens drive anti-tumor immunity in brain tumors, in contrast to its effect in other tumor types. Upon castration, tumor growth was accelerated with attenuated T cell function in GBM and brain tumor models, but the opposite was observed when tumors were located outside the brain. Activity of the hypothalamus-pituitary-adrenal gland (HPA) axis was increased in castrated mice, particularly in those with brain tumors. Blockade of glucocorticoid receptors reversed the accelerated tumor growth in castrated mice, indicating that the effect of castration was mediated by elevated glucocorticoid signaling. Furthermore, this mechanism was not GBM specific, but brain specific, as hyperactivation of the HPA axis was observed with intracranial implantation of non-GBM tumors in the brain. Together, our findings establish that brain tumors drive distinct endocrine-mediated mechanisms in the androgen-deprived setting and highlight the importance of organ-specific effects on anti-tumor immunity.

## Introduction

Sex differences in cancer have been long recognized in non-reproductive organs, such as bladder, colorectal system, lung, skin, and brain^1,2^, and recent efforts have started to highlight the mechanisms underlying these differences. In general, males exhibit a higher incidence and poorer outcomes compared to females across these cancers. Sex hormones and sex chromosomes, including the loss of the Y chromosome^3,4^, are the main factors driving tumor-intrinsic^5^ or tumor-extrinsic^6^ sex differences in cancers. Recently, a crucial role for androgens in anti-tumor immunity and their impact on immune checkpoint inhibitor treatment have been identified. Specifically, inhibition of androgen receptor (AR) signaling enhances the efficacy of anti-PD1 treatment in mouse models of castration-resistant prostate cancer^7^, bladder cancer^8^, and colon cancer^9^. This is likely due to the increased T cell exhaustion induced by AR^8,9^. Therefore, blocking AR can synergize with anti-PD1/PD-L1 blockade to reinvigorate T cell function.

Glioblastoma (GBM) is the most common and malignant primary brain tumor. GBM also displays sex differences, with poorer outcomes observed in males^10,11^, sparking efforts to identify mechanisms, including tumor-intrinsic factors^12,13^ and tumor microenvironment factors^14–16^, underlying these sex differences. Androgen-mediated regulation of tumor growth has been reported in GBM as well. AR signaling can enhance proliferation, migration, and invasion of GBM cells *in vitro*^17–19^. Additionally, *in vivo* studies using immunodeficient mouse models and non-orthotopic transplantation found that loss of androgen signaling inhibited tumor growth in GBM models^18,19^. Based on these findings, AR blockade has been suggested as a potential therapy for GBM. However, the comprehensive effect of androgens on GBM, especially the involvement of the immune compartment, has not been fully addressed.

Androgens play a crucial role in shaping the sexual differentiation of the brain, particularly during the prenatal period^20^, which is more prominent compared to that of almost all other non-reproductive organs. Androgen signaling masculinizes the male brain by guiding the development of male-typical neural structures and functions, which regulates behavioral characteristics of males^20,21^. Cytochrome P450 (aromatase) that converts androgens to estrogen, is highly expressed in the brain, and its expression level varies in different brain regions, contributing to the unique regulation of androgen signaling in the brain^22,23^. Considering the distinctive nature of androgen signaling in the brain, it is likely that androgen signaling contributes in some capacity to the sex differences observed in brain tumors, including GBM. Here, we explored the role of androgens in anti-tumor immunity in GBM and found that, in combination with the loss of AR signaling, brain tumors uniquely regulate immune responses via the hypothalamus-pituitary-adrenal gland (HPA) axis. Moreover, our findings demonstrate that androgens function as an immune-based tumor suppressor in brain tumors and underscores the distinctive immune microenvironment of the brain.

## Results

### T cell abundance negatively correlates with age only in male GBM patients

We previously demonstrated a male-biased increase in T cell exhaustion in GBM that contributes to worse outcomes in male patients^15^. To further understand the sex differences in anti-tumor immunity that occur with aging, we analyzed T cell abundance in tumor samples obtained from 58 patients with high grade gliomas (22 females, 36 males) using image-localized biopsies^24^. Bulk RNA sequencing on tumor samples was performed, and data was deconvolved to estimate T cell abundance in each sample. As there were multiple samples per patient, the average value of the T cell abundance per patient was obtained and presented for statistical analysis. Biopsies of male patients older than 50 years old at diagnosis showed a significant decrease in T cell abundance (*p*=0.041) compared to those younger than 50 (**Extended Data Fig. 1A**). Meanwhile, age had no effect on T cell abundance in biopsies from female patients (*p*=0.69) (**Extended Data Fig.1B**). While multiple biological processes can exert age-dependent effects on tumor progression^25^, we were particularly interested in whether the gradual reduction in sex hormones with aging play a role^26^. Thus, we questioned whether the decreased androgen levels in males had an impact on regulating anti-tumor immunity in brain tumors.

### Castration leads to shortened survival of male mice in intracranial implantation models

**Figure 1.**
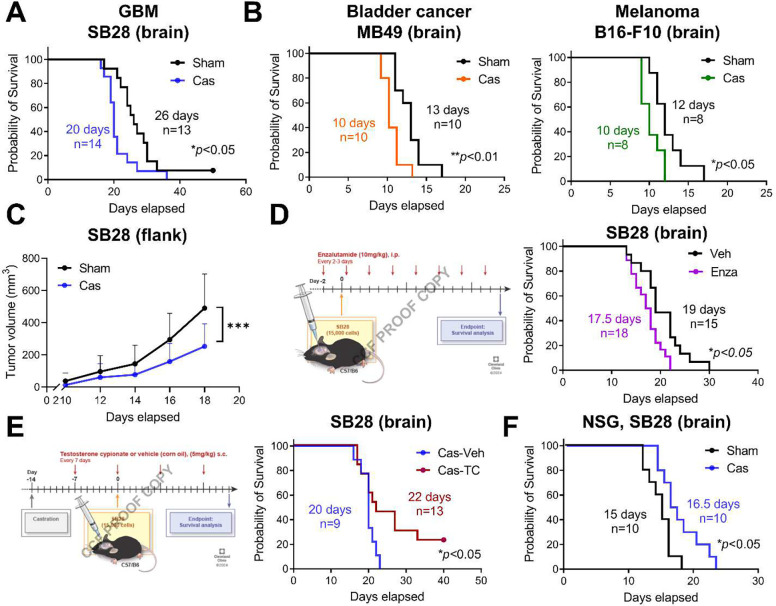
Loss of testosterone leads to shortened survival of brain tumor-bearing mice in an androgen-dependent manner. **A**-**B**. Kaplan-Meier curve depicting survival of B6 mice after intracranial implantation of (**A**) SB28 cells (15,000 cells/mouse) or (**B**) MB49 (5,000 cells/mouse) or B16-F10 (40,000 cells/mouse). **C**. Tumor growth curve of B6 mice inoculated with SB28 subcutaneously in the flank region. Data combined from two independent experiments. n=10/sham, n=9/cas. Data represent the mean ± SD analyzed by two-way ANOVA with Tukey’s multiple comparison test for tumor growth (***p<0.001). **D.** Survival analysis of B6 male mice intracranially implanted with SB28 cells after enzalutamide treatment as depicted. **E**. Survival analysis of castrated B6 male mice intracranially implanted with SB28 cells after testosterone cypionate injections (TC, 250 μg/injection, s.c. weekly) or vehicle (veh, corn oil). **F**. Kaplan-Meier curve depicting survival of NSG mice after intracranial implantation of SB28 cells (10,000 cells/mouse). For survival analysis, median survival days and number of animals are indicated in the graph. Data combined from two to three independent experiments. Log-rank test was performed (*p<0.05, **p<0.01).

To investigate the effect of androgens in brain tumor progression, 5–6-week-old male mice were surgically castrated and survival was analyzed following intracranial implantation of the murine GBM models SB28 and GL261. Unlike other tumor models where castration increased survival^8,9,27^, the survival of castrated mice harboring intracranial GBM implants was significantly decreased compared to the sham group (Fig. 1A, **Extended Data Fig. 2A**). These results indicate that androgens may function as a tumor suppressor in brain tumors, prompting us to question whether this is a tumor cell-specific or site-specific effect. To address this question, we intracranially implanted non-GBM models of bladder cancer (MB49) and melanoma (B16-F10). In these tumor models, immunosuppressive and tumor-promoting roles for androgens were previously demonstrated^8,9^. In both tumor models, we observed shortened survival in castrated mice (Fig. 1B), suggesting that the tumor-suppressive role of androgens is more likely site specific. Furthermore, when murine GBM cells (SB28) were subcutaneously implanted, tumor growth was delayed in the castration group (Fig. 1C). This observation aligns with previous reports on other tumors^8,9^ and supports the brain-specific effect of androgens. Collectively, these results suggest that loss of androgens plays a distinct role in controlling tumor growth when tumors are present in the brain.

To confirm that the decreased survival in castrated mice with a brain tumor is an androgen-dependent effect, we treated gonadally intact male mice (8–9 weeks old) with enzalutamide, an androgen receptor blocker widely used in prostate cancer treatment^28^. Enzalutamide-treated mice showed decreased survival compared to the vehicle treatment group (Fig. 1D), suggesting that androgen receptor signaling mediates this survival difference. Furthermore, administration of exogenous testosterone extended survival in castrated mice, rescuing the decrease in survival observed with castration (Fig. 1E).

To assess the direct effects of androgens on GBM growth, we utilized *in vitro* and immunodeficient *in vivo* models. As previously shown^17–19^, addition of testosterone cypionate to murine GBM cell cultures significantly increased tumor cell number (**Extended Data Fig. 2B**), while both GBM models (SB28, GL261) expressed high levels of AR (**Extended Data Fig. 2C**). Consistently, in immunodeficient NSG mice with intracranial injection of SB28 cells, a delay in tumor growth was observed upon surgical castration compared to the sham-surgery group (Fig. 1F). These data indicate that tumor-extrinsic factors, specifically the immune compartment, play a role in delivering the tumor-suppressive effect of androgens in brain tumor models.

### Brain tumors drive systemic immunosuppression in the absence of androgens

**Figure 2.**
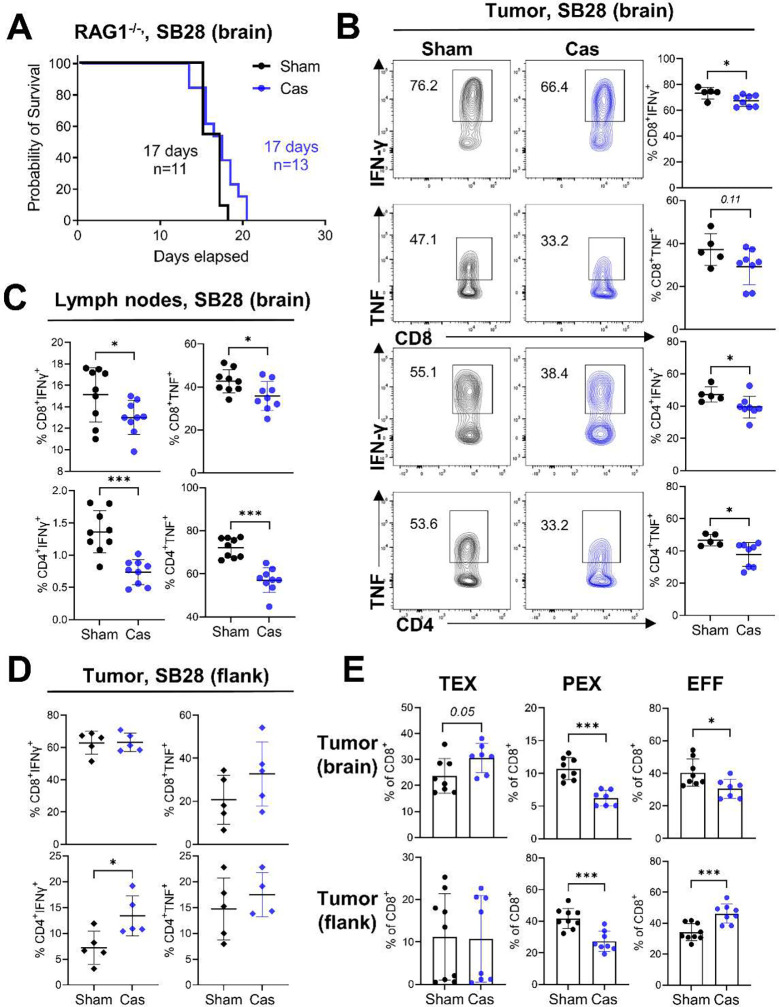
Castration induces a systemic attenuation of T cell function. **A**. Survival analysis of RAG1^−/−^ mice after castration or sham surgery with a brain tumor. Combined results from four independent experiments with log-rank test. Median survival length and number of animals are indicated. **B-C**, Flow cytometric analysis of T cells was performed in sham or castrated mice 14 days after brain tumor implantation. Cytokine production in T cells infiltrated into (**B**) tumors (n=5/sham, n=8/cas) or (**C**) inguinal lymph nodes (n=9/group) was measured after a 4 h incubation with stimulation cocktail. **D**. Cytokine production in T cells infiltrated into flank tumors (n=5/group). **E**. Frequency of exhausted T cell subsets in CD8^+^ T cells from brain tumors (upper) (n=8/sham, n=7/cas) or flank tumors (lower) (n=9/sham, n=8/cas). Terminally exhausted: CD8^+^CD44^+^PD1^+^TIM3^+^TCF1^−^, Progenitor exhausted: CD8^+^CD44^+^PD1^+^TIM3^−^TCF1^+^, Effector: CD8^+^CD44^+^TIM3^−^TCF1^−^. Data combined from two independent experiments. Unpaired Student’s *t*-test was performed (*p<0.05, **p<0.01, ***p<0.01).

Next, we sought to mechanistically understand how the loss of androgens leads to opposing effects on controlling tumor growth in brain, compared to non-brain tumor models. To investigate the role of immune cells, we employed immunocompromised RAG1^−/−^ mice. The castration effect on survival was abrogated in RAG1^−/−^ mice after intracranial implantation of GBM cells (Fig. 2A, **Extended Data Fig. 3A**), suggesting that adaptive immunity and lymphocytes play a critical role in mediating castration effects on survival.

Immune cell profiling revealed that the production of anti-tumor cytokines, such as IFN-γ and TNF-α, was significantly decreased in castrated mice, not only in tumor-infiltrating T cells (Fig. 2B, **Extended Data Fig. 3B**) but also in peripheral lymphoid organs such as lymph nodes (Fig. 2C) and spleen (**Extended Data Fig. 3C**). No difference in the frequencies of immune cell subsets infiltrated into the tumor was observed (**Extended Data Fig. 3D**). This decreased T cell function could explain the accelerated tumor growth in castrated mice. These findings contrast with those in recent publications in other solid tumors, where enhanced T cell function was observed with surgical castration^8,9,27^. Indeed, in flank tumor model with subcutaneous implantation of GBM cells, castration resulted in either comparable or increased T cell function in tumors (Fig. 2D), with elevated CD8^+^ T cell tumor infiltration (**Extended Data Fig. 3E**). Interestingly, in both brain and flank tumor models, an elevated frequency of progenitor exhausted T cells (PEX) was observed in castrated mice (Fig. 2E), supporting the previous findings on AR-mediated regulation of TCF1 transcription^8^. Alternatively, terminally exhausted T cells (TEX) and the effector T cell population (EFF) showed opposite patterns between brain tumors and flank tumor (Fig. 2E). While the TEX population was increased in the castration group with brain tumors, EFF cells were decreased in the brain tumors, but increased in the flank tumors (Fig. 2E). These data indicate additional mechanisms of regulating T cell function in brain tumors under the castration condition beyond androgen-mediated regulation of T cells. Collectively, these results demonstrate that loss of androgens induces systemic T cell dysfunction in a brain tumor-specific manner, which ultimately impacts tumor growth.

### Increased serum glucocorticoids lead to shorted survival upon castration

**Figure 3.**
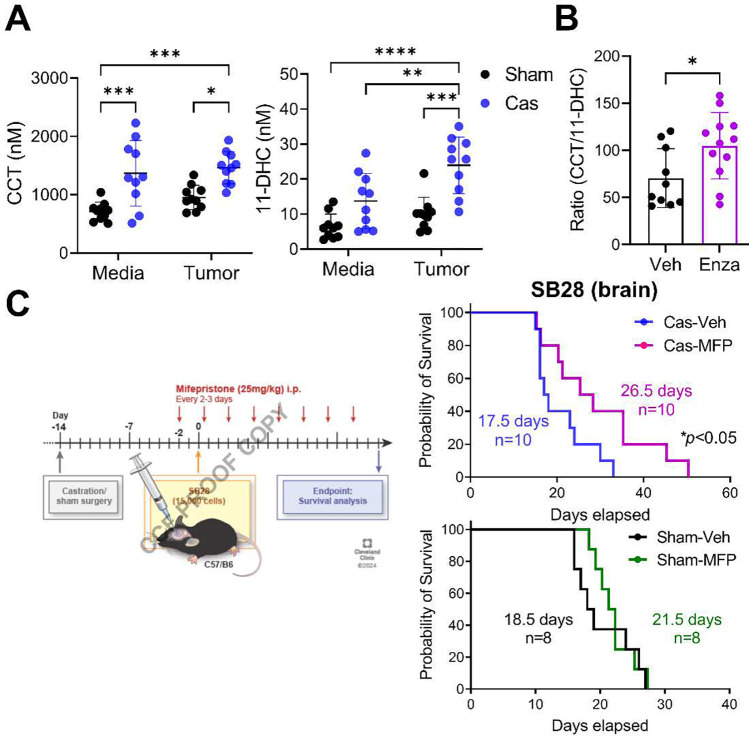
Elevated serum glucocorticoid levels result in reduced survival following castration. **A**. Mouse serum was collected 14 days after intracranial tumor implantation or media injection. Mass spectrometry analysis was performed to measure the levels of corticosterone (CCT) and 11-DHC (11-dehydrocorticosterone). n=10/group. Two-way ANOVA analysis with Tukey’s multiple comparison test was performed (*p<0.05, **p<0.01, ***p<0.001). **B**. Ratio of CCT/11-DHC measured in serum of mice treated with enzalutamide (Enza, 10 mg/kg, i.p.) or vehicle (Veh, corn oil) three times a week. n=10/veh, n=12/enza. **C**. Survival analysis of mice bearing a brain tumor (SB28) and treated with mifepristone (MFP, 25 mg/kg, i.p.) or vehicle (Veh, corn oil) three times a week. Median survival length and number of animals are indicated in the graph. Data combined from two independent experiments. Experiments for castration (upper) and sham (lower) mice were performed separately. Long-rank test (*p<0.05).

Our findings thus far show that testosterone functions to constrain brain tumor growth, the opposite phenotype compared to what has been observed in other solid cancers. Given the systemic immunosuppression we observed (shown in Fig. 2) and acknowledging the well-established role of stress hormones, such as glucocorticoids, in decreasing T cell function^29^, we investigated whether the effects we detected could be attributed to changes in endogenous glucocorticoid levels. Indeed, liquid chromatography-mass spectrometry (LC/MS) analysis of serum from castrated mice revealed a significant elevation of both corticosterone (CCT), an active form of glucocorticoid, and its inactive form, 11-dehydrocorticosterone (11-DHC) (Fig. 3A). Of note, the increase in CCT was observed regardless of brain tumor presence, whereas 11-DHC was further increased by the presence of a tumor in castrated mice (Fig. 3A). The decreased testosterone in castrated mice was confirmed by mass spec analysis (**Extended Data Fig. 4A**). Upon AR blockade with enzalutamide, the serum concentration of glucocorticoids as well as testosterone was not altered (**Extended Data Fig. 4B**). However, the ratio of active to inactive form (CCT/11-DHC) was significantly higher in AR-blocked mice (Fig. 3B), as previously reported in enzalutamide-treated prostate cancer patients^30^ These data suggest that an additional mechanism regulating glucocorticoid metabolism is altered upon pharmacological blockade of AR.

Next, we investigated whether an increase in glucocorticoids contributes to the shortened survival in castrated mice by blocking glucocorticoid receptor (GR) during tumor progression using mifepristone (MFP). The blockade of GR significantly extended the survival of castrated mice compared to the vehicle-treated castration group (Fig. 3C, **upper graph**). This treatment effect was not observed in the sham group (Fig. 3C, **lower graph**), suggesting that increased endogenous glucocorticoids upon castration play a critical role in controlling tumor progression.

### Hypothalamus-pituitary-adrenal (HPA) axis activation is exacerbated by brain tumors upon castration

**Figure 4.**
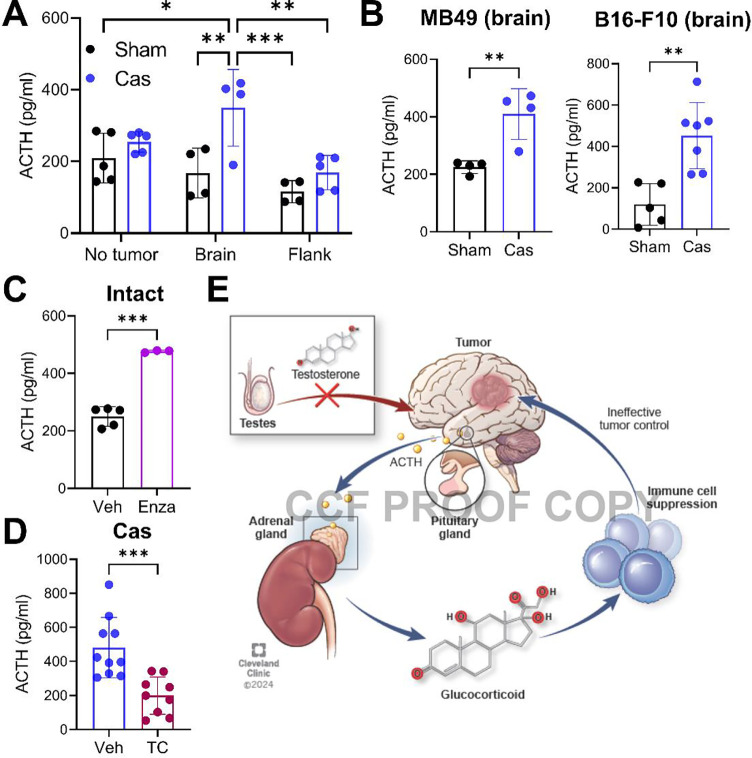
Castration-induced activation of the HPA axis is exacerbated by presence of a brain tumor. **A.** Serum ACTH level was measured using ELISA. Serum was collected 14 days (brain) or 20 days (flank) after SB28 tumor implantation or from mice without a tumor. n=4–5/group. Two-way ANOVA with Tukey’s multiple comparison test (*p<0.05, **p<0.01, ***p<0.001). **B**. Serum ACTH level from mice after intracranial tumor implantation with MB49 (5,000 cells/mouse) or B16-F10 (40,000 cells/mouse). Unpaired student *t*-test (*p<0.05, **p<0.01). **C**. Serum ACTH level from gonadally intact SB28-bearing (brain) mice treated with vehicle (Veh, corn oil) or enzalutamide (Enza, 10 mg/kg, i.p.). Serum samples were collected at endpoint. n=5/veh, n=3/enza. **D**. Serum ACTH level from castrated SB28-bearing (brain) mice treated with vehicle (Veh, corn oil) or testosterone cypionate (TC, 250 μg/injection, s.c., weekly). Serum samples were collected at endpoint. n=10/veh, n=9/TC. Unpaired *t*-test (*p<0.05, ***p<0.001). **E**. Proposed model depicting how the loss of testosterone and the presence of a brain tumor synergistically activate the HPA axis and regulate anti-tumor immunity.

Glucocorticoid production is tightly controlled by the neuro-endocrine system via the HPA axis^31^. As production of glucocorticoids in the adrenal gland is regulated by adrenocorticotropic hormone (ACTH) produced by the pituitary gland, we measured the levels of ACTH in the serum. While no difference was found between the sham and castration groups without intracranial or flank tumor implantation, a significant rise in ACTH level was observed in castrated mice with a brain tumor (Fig. 4A). Additionally, intracranial implantation of non-GBM cells led to the similar elevation of ACTH level upon castration, indicating that the elevation of ACTH is not GBM specific, but site specific (Fig. 4B). The increase in ACTH was dependent on AR signaling, as treatment with AR blocker in brain tumor-bearing mice also resulted in increased ACTH production (Fig. 4C). Furthermore, the increased production of ACTH was reversed by exogenous testosterone treatment in castrated mice (Fig. 4D). Taken together, our data suggest that brain tumors induce hyperactivation of the HPA axis in the absence of androgen signaling, which may lead to decreased anti-tumor T cell immunity and ultimately exacerbate tumor progression (Fig. 4E).

## Discussion

In this study, we demonstrated that loss of androgens alters of the HPA axis in the presence of a brain tumor, in turn inducing immunosuppression and ultimately leading to accelerated tumor progression. While immunotherapies have revolutionized treatment of certain cancers, clinical trials of immunotherapy for GBM have been unsuccessful^32,33^. This could be due to tumor-intrinsic factors, such as the high heterogeneity and low mutational burden in GBM^34,35^. Moreover, the immunologically cold feature of GBM is attributed in part to its location, which includes the blood-brain barrier and brain-resident myeloid cells, microglia, and infiltrating immunosuppressive myeloid cells^36^. Therefore, understanding the unique immune environment and response in the brain is crucial for developing effective treatment for GBM. Recent publications in cancer immunology have highlighted the suppressive role of androgens on anti-tumor T cell responses as well as on the efficacy of immune checkpoint inhibitor therapies^7–9^. Our findings contrast with these other studies in that the loss of androgen signaling negatively impacted anti-tumor immunity as well as disease outcomes. Mechanistically, we found that the neuroendocrine system, specifically the HPA axis, was altered in response to the presence of a brain tumor when androgen signaling was absent. Considering the complexity of brain structures and their role in regulating a variety of functions, our findings underscore the importance of understanding the brain in the context of tumor biology.

The HPA response to stress shows sex differences, with females typically displaying a more pronounced activation of the stress response compared to males^37^. These sex differences primarily arise from activating effects of circulating sex hormones or are patterned during *in utero* development^38^. The inhibitory effect of androgens on the HPA axis has been well documented in the context of stress. Studies have shown that castration in male rats induced a stronger stress response, and this was reversed by exogeneous testosterone treatment^39,40^. In this study, we demonstrated similar findings in our brain tumor models, emphasizing that crosstalk between gonadal and adrenal hormones can impact tumor outcomes. However, the mechanism by which brain tumors induce stress responses remains unclear. Proinflammatory cytokines such as IL-1β, IL-6, and TNF can directly activate the HPA axis and trigger production of glucocorticoids^41–43^. Given that these proinflammatory cytokines are produced during brain tumor progression^44^, it is possible that neuroinflammation may contribute to the hyperactivation of the HPA axis. In addition, the interaction between neurons and tumors has recently become recognized in a variety of cancers^45^, particularly in brain tumors including GBM^46,47^. Considering the neuron-rich environment of the brain, it is plausible that tumors interact with neurons in the hypothalamus and trigger downstream stress responses. Future studies will focus on elucidating the underlying mechanisms by which brain tumors stimulate the HPA axis.

The median age at diagnosis for GBM is 68–70 years old^48^, and age negatively impacts the patient survival^49^. Given that serum testosterone production in men decreases with age^26^, it is crucial to consider the potential impact of diminished sex hormones in male patients. While our patient data suggest a negative impact of aging on T cell abundance in male GBM samples (**Extended Data Fig. 1**), it will be necessary to assess the effect of androgen levels in GBM patients on their clinical outcomes. A limitation of the current study involves the use of young male mice (5–6 weeks old), which does not reflect age-related changes in immune system and endocrine functions. Thus, future studies will focus on evaluating the effect of androgens on brain tumors within the context of aging using appropriate animal models. Meanwhile, GBM patients are often treated with dexamethasone for edema control, especially around the time of surgery and radiation therapy^50^. Dexamethasone potently suppresses immunity, inflammation, and the HPA axis. Thus, the combined effect of decreased serum testosterone and dexamethasone requires further consideration in a clinical setting. Taken together, our findings highlight the distinct combined effects of brain tumors and decreased androgen signals on anti-tumor immunity and tumor control. This underscores the significance of comprehending brain tumor biology, given its unique anatomical and functional location.

## Materials and Methods

### Cell lines

The syngeneic mouse GBM cell model SB28 was generously provided by Dr. Hideho Okada (University of California San Francisco), and GL261 was obtained from the Developmental Therapeutic Program, NCI. The murine bladder cancer cell line MB49 was obtained from the Animal Tumor Core at the Cleveland Clinic. The murine melanoma B16-F10 cells were kindly gifted by Dr. Thaddeus Stappenbeck (Cleveland Clinic). Upon thawing, all cell lines were treated with 1:100 MycoRemoval Agent (MP Biomedicals) and regularly tested for Mycoplasma spp. (Lonza). GBM cell lines were maintained in complete RPMI 1640 (Media Preparation Core, Cleveland Clinic) supplemented with 10% FBS (Thermo Fisher), 1% penicillin/streptomycin (Media Preparation Core), and GlutaMAX (Gibco). MB49 and B16-F10 cells were cultured in DMEM (Media Preparation Core, Cleveland Clinic) supplemented with 10% FBS, 1% penicillin/streptomycin, GlutaMAX, and sodium pyruvate (Thermo Fisher Scientific). Cells were cultured in humidified incubators at 37°C and 5% CO^2^ and were not allowed to exceed 15 passages.

### Mice

C57BL/6 (JAX: 000664), RAG1^−/−^ (JAX: 002216; B6.129S7-Rag1tm1Mom/J), LCK-cre (JAX: 003802; B6.Cg-Tg(Lck-cre)548Jxm/J), Foxp3-cre (JAX: 016959; B6.129(Cg)-Foxp3tm4(YFP/icre)Ayr/J), and GR-flox (JAX: 021021; B6.Cg-Nr3c1tm1.1Jda/J) mice were purchased from The Jackson Laboratory as required. NSG mice were obtained from the Biological Resource Unit (BRU) at Lerner Research Institute, Cleveland Clinic. All animals were kept in a specific pathogen-free facility of the BRU, with a 12-hour light-dark cycle. All animal procedures were performed in accordance with the guidelines and protocols approved by the Institutional Animal Care and Use Committee (IACUC) at the Cleveland Clinic.

### Castration

Two weeks prior to tumor implantation, 5- to 6-week-old male mice underwent either castration or sham surgery. Mice were maintained under inhalation anesthesia (2–2.5% isoflurane) through a nose cone and administered an ophthalmic lubricant to prevent corneal dryness. The scrotal area was disinfected using betadine and alcohol. A small horizontal incision was made in the skin of the scrotum and the inner skin membranes, and the testicles were exteriorized. Using resorbable vicryl sutures, testicular arteries were ligated, followed by the removal of testicles. The incision was closed using surgical clips (Fine Science Tools). For pain control, subcutaneous injections of buprenorphine (0.1 mg/kg) and bupivacaine (5 mg/kg) were administered. In sham-operated mice, the same procedure was performed, excluding the ligation and removal of the testis.

### Tumor implantation and treatments

For intracranial tumor implantation, mice were anesthetized by inhalation anesthesia (2–2.5% isoflurane), secured in the stereotaxis apparatus, and intracranially injected with tumor cells suspended in 5 μl RPMI-null media. The injection was targeted to the left hemisphere, approximately 0.5 mm rostral and 1.8 mm lateral to the bregma with a depth of 3.5 mm from the scalp. The needle was held in place an additional 60 seconds before a slow and measured removal. The animals were monitored to detect the onset of neurological and behavioral symptoms indicative of the presence of a brain tumor. For subcutaneous tumor implantation, mice were anesthetized by inhalation anesthesia (2–2.5% isoflurane). A total of 500,000 SB28 cells was suspended in 100 μl RPMI-null media and injected subcutaneously into the right flank region of the mice. Tumor size was measured starting from day 10 when the tumor become palpable, and measurements were taken every 2 days.

In some experiments, gonadally intact male mice received intraperitoneal injections of enzalutamide (10 mg/kg; SellekChem) or vehicle (corn oil) beginning two days before tumor implantation. The injections were repeated every 2 to 3 days until the experimental endpoint was reached. In other experiments, intraperitoneal injections of mifepristone (25 mg/kg; Cayman Chemical) or vehicle (corn oil) were initiated two days prior to tumor implantation and were repeated every 2 to 3 days until reaching endpoint.

To restore testosterone level in castrated mice, testosterone cypionate injections (12.5 mg/kg; Hikma Pharmaceuticals) were given subcutaneously one week prior to tumor implantation and repeated once a week.

### Tumor and tissue dissociation for flow cytometry

At the indicated time, mice were euthanized as described above, and brain tumor, spleen, and lymph nodes (inguinal) were harvested. Brain tumor tissue was minced into small pieces with scalpels and subjected to enzymatic digestion in the presence of collagenase D (1 mg/ml; Roche) and DNase I (0.1 mg/ml; Roche) at 37°C. Digested tissue was filtered through a 70 μm cell strainer. To enrich for immune cells, gradient centrifugation was performed using 30% percoll solution (Sigma). Red blood cells (RBCs) were lysed using RBC lysis buffer (BioLegend). For spleen and lymph nodes, tissue was ground onto a 40 μm cell strainer, followed by RBC lysis. All single-cell suspension samples were filtered once more with a 40 μm cell strainer before staining for flow cytometry.

### Flow cytometry

Cells were stained with the antibodies listed in **Extended Data Table 1&2**. Briefly, after live/dead staining with LIVEDEAD Blue (Thermo Fisher Scientific) on ice for 15 min, cells were washed and incubated with FcR blocker (Miltenyi Biotech) diluted in PBS/2% BSA on ice for 10 minutes. For surface staining, cells were incubated in an antibody mixture diluted in brilliant buffer (BD Biosciences) at 1:100 to 1:250 on ice for 30 minutes. After washing with PBS/2% BSA buffer, cells were fixed with Foxp3/Transcription factor fixation buffer (eBioscience) overnight. For intracellular staining, antibodies were diluted in Foxp3/Transcription factor permeabilization buffer at a ratio of 1:250 to 1:500, and cells were incubated at room temperature for 45 minutes. For intracellular cytokine detection, cells were stimulated using Cell Stimulation Cocktail plus protein transport inhibitor (eBioscience) in complete RPMI for 4 hours, followed by the cell staining procedures described above. Stained cells were acquired with an Aurora (Cytek Biosciences) and analyzed using FlowJo software (v10, BD Biosciences).

### Image-Localized Biopsy Deconvolution and Analysis

A total of 202 biopsies collected from 58 patients (22 females, 36 males) with high-grade glioma^24^ were analyzed for bulk RNA-Seq^51^ and underwent CIBERSORTx deconvolution alongside a snRNA-Seq reference^52^ with clustered cell states as previously described^53^, producing estimates of T cell abundances in each sample. Due to the limited storage available on the CIBERSORTx online interface, snRNA was downsampled 3 times to produce 100 of each cell state as input into the algorithm, each run 6 times. We present an average across runs. Statistics presented for this data are a result of t-test within patient sex. T cell values were averaged within patients not to violate the assumption of independent samples.

### Tumor cell proliferation assessment

Tumor cell proliferation was monitored and quantified using the IncuCyte Live-Cell Analysis System. For these experiments, four technical replicates of SB28 (500 cells/well, 200 μl) and GL261 (1,000 cells/well, 200 μl) were plated in flat-bottom 96-well plates and treated with testosterone cypionate or vehicle (corn oil, 2 μl/well). Data was captured after a 96-hour incubation.

### ACTH ELISA

Serum was collected at the indicated time points or endpoint. ACTH level was measured using the mouse/rat ACTH ELISA kit (abcam) following the manufacturer’s instructions. Serum was diluted at a ratio of 1:2 to 1:4.

### Mass spectrometry

Freshly collected mouse serum samples were stored at −80°C until analysis. Concentration of glucocorticoids and testosterones were measured using LC-MS/MS analysis as previously described^54^. Briefly, 60 μl of thawed serum was spiked with internal standards mix (Androstene-3, 17-dione-2, 3, 4-^13^C_3_ and 5α-Dihydrotestosterone-16, 17, 17-D3 and Cortisol-9, 11, 12, 12-D4). Protein precipitation was followed by adding acetonitrile and the supernatant was collected to extract glucocorticoids and testosterone using methyl-tert-butyl ether through liquid-liquid extraction procedure. The steroids fraction was collected, dried, and reconstituted in 140 μl of 50% methanol. The reconstituted sample underwent LC-MS/MS analysis on a Shimadzu UPLC system with a C18 column (Zorbax Eclipse Plus C_18_ column, 150 mm × 2.1 mm, 3.5 μm, Agilent, Santa Clara, CA), coupled to a QTrap 5500 mass spectrometer (AB Sciex, Redwood City, CA). Data acquisition and processing were performed using MultiQuant (AB Sciex; version 3.0.3).

### Statistical Analysis

GraphPad Prism (Version 9, GraphPad Software Inc.) software was used for data presentation and statistical analysis. Unpaired Student’s *t* test or one-/two-way analysis of variance (ANOVA) was used with Tukey’s multiple comparisons test, as indicated in the figure legends. Survival analysis was performed by the log-rank test. *p*<0.05 was considered statistically significant (*, *p*< 0.05; **, *p*< 0.01; ***, *p*< 0.001).

## Data Availability

All data generated in this study are available upon request from the corresponding author, Dr. Justin D. Lathia (lathiaj@ccf.org).
